# Human Immunodeficiency Virus–Induced Interferon-Stimulated Gene Expression Is Associated With Monocyte Activation and Predicts Viral Load

**DOI:** 10.1093/ofid/ofae434

**Published:** 2024-08-05

**Authors:** Lisa van Pul, Karel A van Dort, Arginell F Girigorie, Irma Maurer, Agnes M Harskamp, Neeltje A Kootstra

**Affiliations:** Amsterdam Institute for Infection and Immunity, Amsterdam, The Netherlands; Experimental Immunology, Amsterdam University Medical Center, University of Amsterdam, Amsterdam, The Netherlands; Amsterdam Institute for Infection and Immunity, Amsterdam, The Netherlands; Experimental Immunology, Amsterdam University Medical Center, University of Amsterdam, Amsterdam, The Netherlands; Amsterdam Institute for Infection and Immunity, Amsterdam, The Netherlands; Experimental Immunology, Amsterdam University Medical Center, University of Amsterdam, Amsterdam, The Netherlands; Amsterdam Institute for Infection and Immunity, Amsterdam, The Netherlands; Experimental Immunology, Amsterdam University Medical Center, University of Amsterdam, Amsterdam, The Netherlands; Amsterdam Institute for Infection and Immunity, Amsterdam, The Netherlands; Experimental Immunology, Amsterdam University Medical Center, University of Amsterdam, Amsterdam, The Netherlands; Amsterdam Institute for Infection and Immunity, Amsterdam, The Netherlands; Experimental Immunology, Amsterdam University Medical Center, University of Amsterdam, Amsterdam, The Netherlands

**Keywords:** HIV, immune activation, innate immune response, ISG, monocyte

## Abstract

**Background:**

Chronic immune activation is one of the hallmarks of human immunodeficiency virus (HIV) pathogenesis. Persistent upregulation of interferons (IFNs) and interferon-stimulated genes (ISGs) has previously been associated with chronic immune activation and HIV progression. Here a longitudinal analysis of the IFN and ISG response during HIV infection was performed to gain insights into the ongoing immune activation during HIV infection.

**Methods:**

IFN and ISG levels were determined using quantitative polymerase chain reaction in peripheral blood mononuclear cells of people with HIV at pre-seroconversion, during acute and chronic HIV infection, and during suppressive antiretroviral therapy (ART).

**Results:**

HIV infection induced the expression of a set of 4 ISGs—RSAD2, ISG15, IFI44L, and IFI27—which remained upregulated during chronic infection. This set of ISGs showed no clear correlations with T-cell activation as determined by co-expression of CD38 and HLA-DR. However, a strong correlation with monocyte activation marker soluble CD163 in serum was found. Furthermore, the expression of this ISG cluster was predictive of viral load before ART initiation and, on ART, expression levels normalized to pre-seroconversion levels.

**Conclusions:**

The results presented here suggests that ISG expression is linked to monocyte activation, possibly driven by viral replication.

In untreated human immunodeficiency virus (HIV) infection, chronic immune activation has been recognized as one of the main drivers behind CD4 T-cell depletion and progression to AIDS [1–4]. High levels of both innate and adaptive immune activation are caused by virus replication as well as coinfections with other pathogens, gut microbial translocation, and immune dysregulation [5–11]. Antiretroviral therapy (ART) can effectively reduce HIV replication and immune activation in people with HIV (PWH); however, even after treatment there is residual immune activation, which has been associated with long-term morbidity [12, 13].

In the acute phase of HIV infection, interferons (IFNs) are among the molecules that are first released [14]. In turn, IFN receptor signaling by IFNs will induce JAK-STAT signaling and eventually this will lead to the transcription of interferon-stimulated genes (ISGs) [14, 15]. The induction of antiviral molecules encoded by ISGs can block viral replication and thus limit viral spread [16–18]. Moreover, the IFN response can direct both innate and adaptive immune responses [15, 19–21].

In the early stages of infection, type I IFN signaling and ISG induction appear to be beneficial to the host and control of viral replication, whereas persistent upregulation of type I IFNs and ISGs is related to chronic immune activation and progression of HIV infection [22–27]. Indeed, studies in sooty mangabeys and African green monkeys, which are a natural host and have nonpathogenic simian immunodeficiency virus (SIV) infection, the elevated ISG expression seen during the acute phase of SIV infection resolves during chronic viral control [22, 24, 28]. Contrarily, SIV disease progression in pathogenic models showed that SIV infection in rhesus macaques is correlated to persistent upregulation of ISGs [22, 24] and a sustained strong type I and II IFN response in Asian pigtailed macaques [28]. In humans, CD4 T-cell depletion as well as disrupted T-cell dynamics have also been associated with elevated ISG expression [26].

Elucidation of the mechanisms that contribute to chronic immune activation could provide new targets to restore the immune imbalance observed in HIV infection. Here we performed a longitudinal analysis of the ISG response in HIV infection to provide further insights into the ongoing immune activation during HIV infection and during suppressive ART. IFN and ISG expression levels in PWH before seroconversion (SC), during the acute and chronic phase of infection, and before and after ART initiation are determined. CD38 and HLA-DR co-expression on T cells was determined as reflection of activation of the adaptive immune system, whereas CXCL10/IP-10 and soluble CD163 (sCD163), which are mainly produced by monocytes upon activation [29, 30], were analyzed in serum as a measure for monocyte activation. Increased levels of activated CD4 and CD8 T cells, sCD163, and CXCL10/IP-10 have been associated with disease progression in HIV infection [1, 29, 31]. Associations between the IFN and ISG expression and biomarkers of disease progression and immune activation were investigated.

## MATERIALS AND METHODS

### Patient Consent Statement

The Amsterdam Cohort Studies (ACS) on HIV and AIDS [32] has been conducted in accordance with the ethical principles set out in the declaration of Helsinki. Participants’ written informed consent was obtained. The study has been approved by the institutional medical ethical committee of the Academic Medical Center (2007_182) and the ethics advisory body of the Sanquin Blood Supply Foundation in Amsterdam.

### Participant Characteristics

Participants of the ACS who entered the cohort seronegative, had an untreated follow-up of at least 6 years, and from whom pre-SC cryopreserved peripheral blood mononuclear cells (PBMCs) were available were selected. Additional samples before and during ART were included. Participants of whom 1 or more time points were missing were excluded from analysis. Absolute CD4 and CD8 counts and plasma viral RNA loads were determined at every study visit using the diagnostic tests available at that time.

Blood donor samples were obtained from the Dutch national blood bank (Sanquin) in Amsterdam, the Netherlands. Blood donors from the Netherlands are actively screened for HIV, hepatitis B, hepatitis C, syphilis, and human T-lymphotropic virus type 1 infection. Individuals aged >70 years or individuals who display high risk behavior for blood-borne infections are excluded from blood donation.

### PBMC Isolation

PBMCs were isolated from blood using Ficoll-Isopaque (GE Healthcare, Chicago, Illinois) density gradient centrifugation. PBMCs were frozen using Iscove's modified Dulbecco’s medium supplemented with 10% dimethyl sulfoxide and 20% fetal bovine serum. Cryopreserved PBMCs were stored in gas phase liquid nitrogen until use.

### Serum Biomarkers

Soluble CD163 and CXCL10/IP-10 concentrations (pg/mL) in serum were determined by enzyme-linked immunosorbent assay (ELISA) using the manufacturer’s protocol (DuoSet ELISA kits, R&D Systems, Minneapolis, Minnesota).

### Flow Cytometry

T-cell activation was determined by assessing the co-expression of CD38 and HLA-DR on CD4 and CD8 T cells. PBMCs were stained for 30 minutes at 4°C in the dark and then washed with phosphate-buffered saline supplemented with 0.5% bovine serum albumin. After fixation with CellFIX (BD Biosciences, Franklin Lakes, New Jersey), fluorescence was measured on the BD FACS Canto II (BD Biosciences) and marker expression was determined using FlowJo software (TreeStar, Ashland, Oregon). The following fluorescent-labeled antibodies were used: CD3: Brilliant Violet 510 (clone OKT3) and CD8: Pacific Blue (clone SK1) (BioLegend, San Diego, California); CD4: R-phycoerythrin (PE)–cyanine dye Cy7 (clone SK3), CD38: PE (clone HB7), and HLA-DR: fluorescein isothiocyanate (clone L243) (BD Biosciences).

### RNA Isolation and Reverse-Transcription Quantitative Polymerase Chain Reaction

RNA was isolated from PBMC samples using the AllPrep mini kit (Qiagen, Hilden, Germany), according to the manufacturer's instructions. Complementary DNA was generated using oligo(dt) primers (Promega, Madison, Wisconsin) and M-MLV reverse transcriptase (Promega). Quantitative polymerase chain reaction (qPCR) was performed on the LightCycler480 (Roche, Basel, Switzerland) using GoTaq qPCR Master Mix (Promega) using the following program: 95°C for 10 minutes; 50 cycles: 95°C for 10 seconds, 58°C for 20 seconds, 72°C for 30 seconds; and melting curve: 95°C for 5 seconds, 65°C for 1 minute followed by 97°C with acquisition every 10°C. The following genes were analyzed: IFN-α, IFN-β, IFN-γ, RSAD2, IRF5, IRF8, NMI, IFI27, IFI44L, and ISG15. Glyceraldehyde 3-phosphate dehydrogenase and β-actin gene expression were used for normalization. Primer sequences are displayed in Supplementary Table 5. Expression levels were calculated using the 2^–ΔΔCt^ method relative to controls without HIV.

### Statistical Analysis

To determine whether measurements changed significantly over time, the Friedman analysis variance test for repeated measurements followed by Dunn post hoc tests were used (IBM SPSS version 28, Armonk, New York). Correlation analyses were performed using Pearson correlation analysis in RStudio (version 4.2.1) [33], and *q* values are given to control for the false discovery rate. Linear regression analysis to examine associations of IFN and ISG expression levels at 5–9 months post-SC with pre-ART biomarkers of disease progression (viral load, CD4 T-cell counts, CD4 T-cell activation, and CD8 T-cell activation) were performed in SPSS. For all analyses, *q* values or *P* values of <.05 were considered to be significant. GraphPad Prism version 9 (San Diego, California) and RStudio were used to generate graphs and correlograms, respectively. The RStudio packages used were corrplot [34] and RColorBrewer [35].

## RESULTS

### Participant Characteristics

Twenty participants from the ACS on HIV and AIDS were included. Participants entered the cohort between 1986 and 1994, before HIV infection, and had a known HIV seroconversion date and started ART during follow-up. Pre-SC and 5–9 months, 2 years, and 6 years post-SC PBMC samples were available for 16 participants while pre- and post-ART PBMC samples were available for 20 participants (Table 1). Absolute CD4 T-cell counts and plasma viral load are shown in Table 1. Increased immune activation has been associated with disease progression in HIV infection [1, 29, 31]. CD4 and CD8 T-cell activation (CD38 and HLA-DR co-expression; Supplementary Figure 1) and soluble markers for inflammation and monocyte activation, sCD163 and CXCL10, at the time of sampling are shown in Table 1.

**Table 1.** Participant Characteristics

Characteristic	No.	Median (IQR)						
Age at SC, y	20	40.7 (35.0–45.3)	…	…	…	…	…	…
Sex (male/female)	20/0	…	…	…	…	…	…	…
Route of HIV infection (MSM/IDU)	18/2	…	…	…	…	…	…	…

	Pre-SC		5–9 mo post-SC		2 y post-SC		6 y post-SC	
	No.	Median (IQR)	No.	Median (IQR)	No.	Median (IQR)	No.	Median (IQR)
Time pre- or post-SC, mo	16	46.3 (33.0–61.0)	16	7.3 (6.7–8.3)	16	24.8 (24.3–27.5)	16	79.8 (69.2–98.8)
CD4 count, ×10^9^ cells/L	16	0.92 (0.77–1.21)	16	0.57 (0.48–0.76)	16	0.50 (0.40–0.66)	16	0.37 (0.28–0.46)
CD8 count, ×10^9^ cells/L	16	0.65 (0.54–0.84)	16	0.79 (0.48–0.91)	16	0.77 (0.57–0.98)	16	1.00 (0.70–1.30)
RNA viral load, copies/mL	…	…	16	14 000 (3350–35 000)^a^	16	13 923 (3100–68 000)^b^	16	12 983 (1767–49 000)
CD4 activation, %	16	1.1 (0.8–1.2)	16	2.0 (1.1–3.1)	16	2.5 (2.0–3.8)	16	4.4 (3.4–6.3)
CD8 activation, %	16	2.4 (1.4–3.4)	16	10.9 (8.0–14.9)	16	11.5 (6.4–19.8)	16	12.6 (7.8–19.6)
Soluble CD163, pg/mL	14	337.2 (234.1–394.3)	16	473.42 (307.3–825.1)	…	…	…	…
CXCL10, pg/mL	14	65.8 (35.2–118.3)	16	207.2 (142.3–384.4)	…	…	…	…

	Pre-ART		Post-ART					
	No.	Median (IQR)	No.	Median (IQR)				
Time pre or post-ART, mo	20	2.0 (0.9–5.2)	20	36.1 (33.0–38.8)	…	…	…	…
CD4 count, ×10^9^ cells/L	20	0.37 (0.28–0.46)	20	0.51 (0.34–0.72)	…	…	…	…
CD8 count, ×10^9^ cells/L	20	1.00 (0.73–1.55)	20	1.02 (0.72–1.56)	…	…	…	…
RNA viral load, copies/mL	20	18 200 (1789–70 000)	20	400 (400–400)^c^	…	…	…	…
CD4 activation, %	20	5.5 (3.5–10.5)	20	2.5 (1.5–3.9)	…	…	…	…
CD8 activation, %	20	13.2 (8.2–18.4)	20	5.4 (2.5–9.3)	…	…	…	…

Abbreviations: ART, antiretroviral therapy; HIV, human immunodeficiency virus; IDU, injection drug user; IQR, interquartile range; MSM, men who have sex with men; SC, seroconversion.

^a^Two individuals with undetectable load (<1000 copies/mL).

^b^Three individuals with undetectable load (<1000 copies/mL).

^c^Sixteen individuals with undetectable load (<400 copies/mL).

### Longitudinal IFN and ISG Expression in Untreated PWH

Total PBMCs from PWH were analyzed for the following IFNs and ISGs by qPCR: type I IFN-α and IFN-β, which are in blood, predominantly produced by monocytes/macrophages and plasmacytoid dendritic cells (pDCs); type II IFN-γ, produced by T cells; and a selection of ISGs (IRF5, ISG15, RSAD2, IFI27, IFI44L, NMI, and IRF8) that have previously been shown to change during acute HIV-1 infection and in pathogenic SIV infection [22, 36–38].

**Figure 1.**
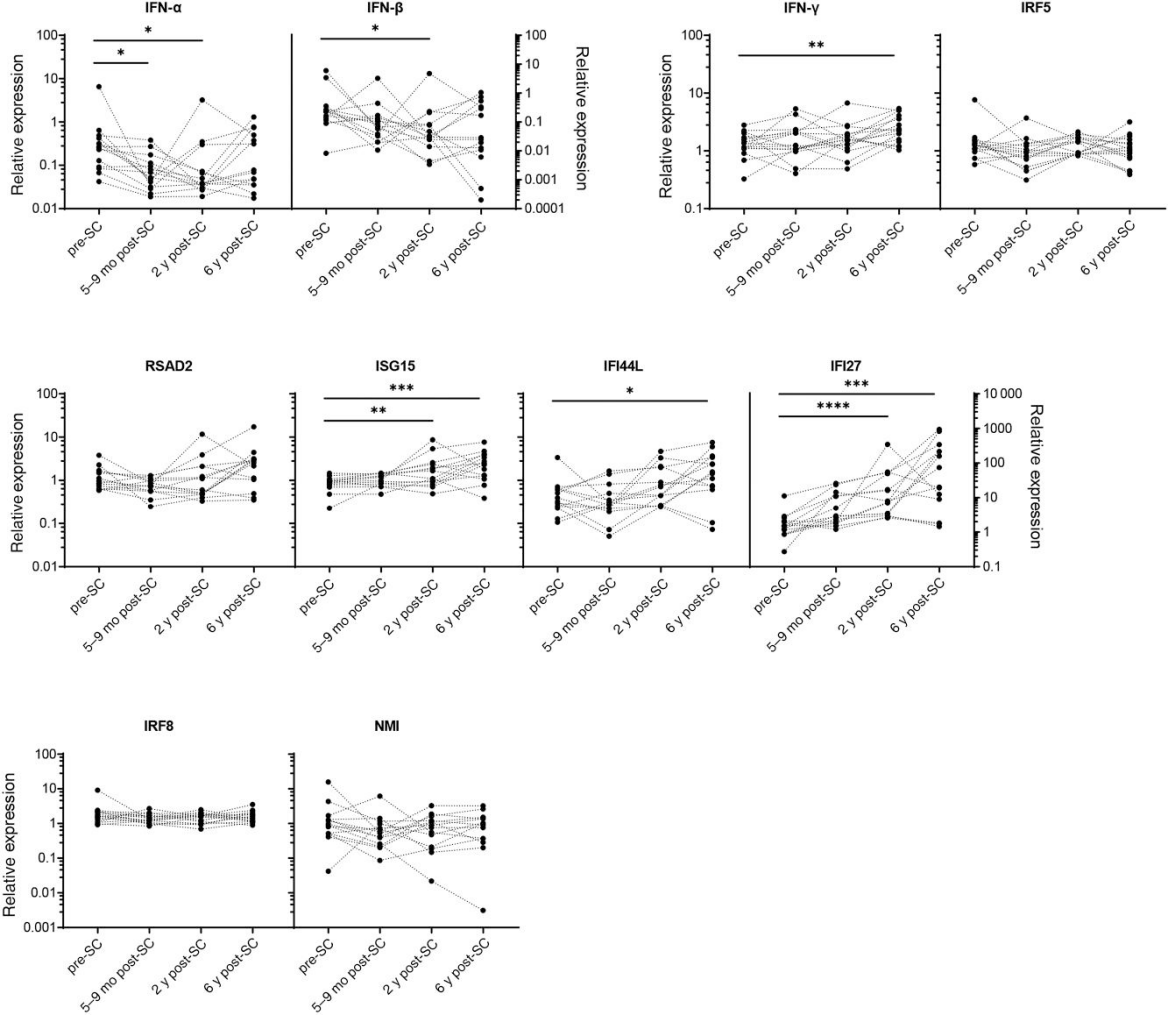
Longitudinal interferon (IFN) and interferon-stimulating gene (ISG) expression levels during human immunodeficiency virus (HIV) infection in people with HIV (PWH). Depicted are the IFN and ISG expression levels in PWH before infection with HIV (pre-seroconversion [SC]) and at 3 time points after HIV infection (5–9 months, 2 years, and 6 years post-SC). Expression levels of ISGs were calculated using the 2^–ΔΔCt^ method relative to controls without HIV. Significant *P* values, as determined by Friedman analysis of variance test for repeated measurements within an individual followed by Dunn post hoc tests: *P < .05; **P < .01; ***P < .001; ****P < .0001. Abbreviations: ART, antiretroviral therapy; SC, seroconversion; sCD163, soluble CD163.

As compared to the pre-SC samples, IFN-α expression levels decreased already at 5–9 months post-SC and similarly low expression levels were observed during follow-up in the majority of the PWH, whereas IFN-α expression levels had recovered at 6 years post-SC in 6 of the PWH (Figure 1). IFN-β expression levels also decreased after HIV infection, albeit at a slower rate (Figure 1). Like IFN-α, recovery of IFN-β expression levels was observed in part of the PWH (n = 7) at 6 years post-SC. IFN-γ expression levels did not change early after HIV infection (5–9 months and 2 years post-SC), but a significant increase as compared to pre-SC was observed 6 years post-SC, during the chronic phase of the disease (Figure 1). ISG15 and IFI27 expression significantly increased after HIV infection starting at the early time point 5–9 months post-SC (Figure 1), whereas increased IFI44L and RSAD2 expression was observed at 2 and 6 years post-SC; however, this increase was only significant for IFI44L during the chronic phase of infection (Figure 1). The expression of IRF5, IRF8, and NMI did not significantly change after HIV infection (Figure 1).

**Figure 2.**
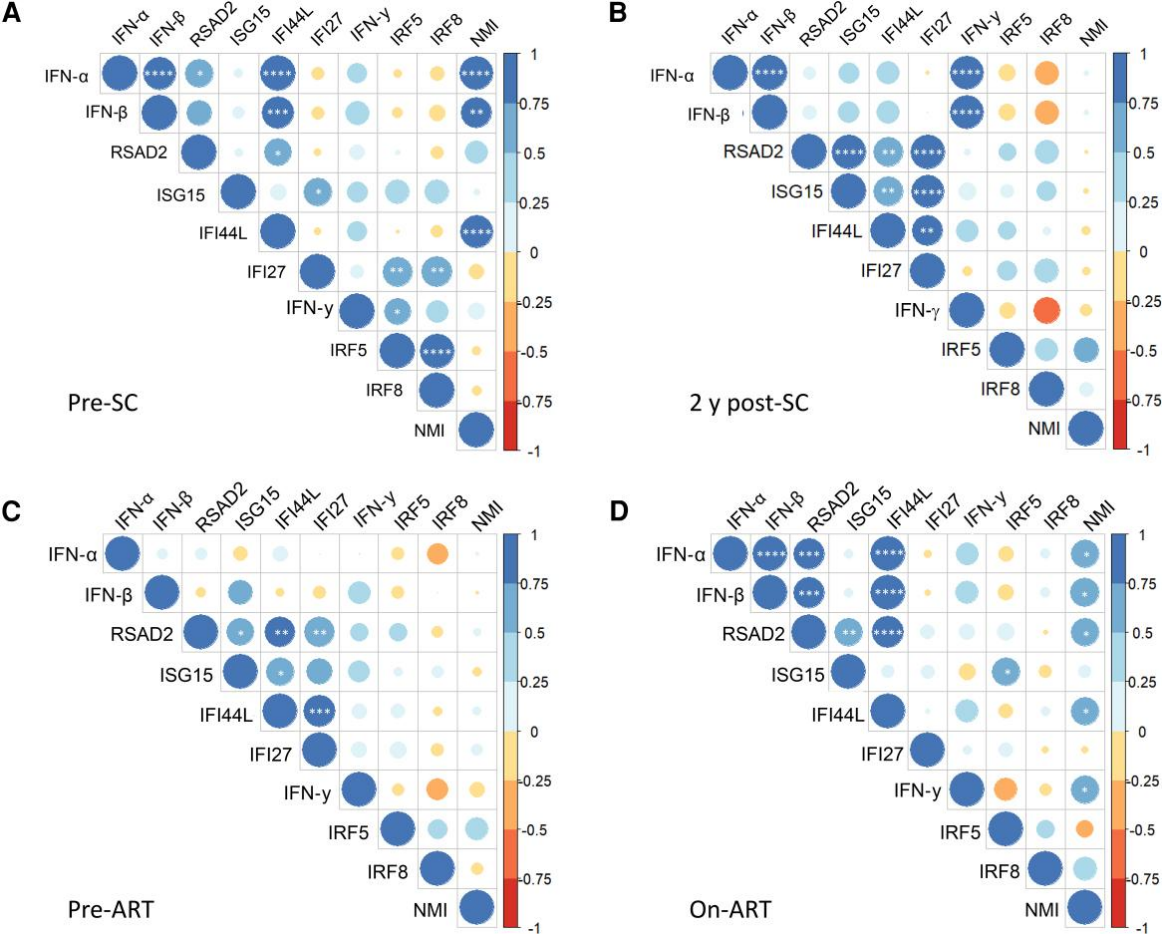
Correlograms of interferon (IFN) and interferon-stimulating gene (ISG) expression levels before and during human immunodeficiency virus (HIV) infection. Depicted are the correlations between IFN and ISG expression levels in people with HIV pre-seroconversion (SC; *A*), 2 years post-SC (*B*), pre–antiretroviral therapy (ART) (*C*), and on ART (*D*). Positive correlations are displayed in blue and negative correlations in red. The color intensity and the size of the circle are proportional to the correlation coefficients. Significant *q* values, as determined by Pearson correlation and adjusted for false discovery rate: *q < 0.05; **q < 0.01; ***q < 0.001; ****q < 0.0001.

Pearson correlation analysis of the ISG expression levels revealed significant positive correlations between IFN-α, IFN-β, RSAD2, IFI44L, and NMI and between IRF5, IRF8, and IFI27 at pre-SC (Figure 2*A*), which were lost after HIV infection (Figure 2*B* and Supplementary Figure 2). At 5–9 months post-SC, a strong positive correlation between RSAD2, ISG15, IFI44L, and IFI27 was observed, which remained present at later time points during chronic phase of HIV infection (Figure 2*B* and Supplementary Figure 2). In addition, there was a positive correlation between NMI, IFN-γ, and IFI44L at 5–9 months post-SC, which was also observed at 6 years post-SC (Supplementary Figure 2). Furthermore, a positive correlation between IFN-α, IFN-β, and IFN-γ was observed at 2 years post-SC, and this cluster was negatively correlated with IRF8 expression, although this was only significant (Figure 2*B*).

Here we show upregulation of a cluster of ISGs (RSAD2, ISG15, IFI44L, and IFI27) upon HIV infection in PWH. The expression of these ISGs remained high during the untreated course of disease.

### IFN and ISG Expression in PWH After ART Initiation

Next, the effect of ART on expression levels of IFNs and ISGs was determined in 20 ACS participants. No significant changes in the expression levels of IFN-α, IFN-β, IFN-γ, and IRF5 were observed after ART initiation (Supplementary Figure 3*A–D*). Upon ART initiation, expression levels of ISG15, IFI27, IFI44L, and RSAD2 decreased to near pre-SC levels (Supplementary Figure 3*E–H*). IRF8 levels remained stable after ART, while NMI expression further declined after ART initiation and was lower as compared to pre-SC levels (Supplementary Figure 3*I–J*).

Pearson correlation analysis showed high similarity between the pre-ART ISG profile (Figure 2*C*) and the ISG profile obtained at 2 and 6 years post-SC (Figure 2*B* and Supplementary Figure 2), with a strong positive correlation between RSAD2, ISG15, IFI44L, and IFI27. This correlation cluster was lost after ART initiation (Figure 2*D*). The on-ART correlogram showed high resemblance to the pre-SC correlogram (Figure 2*A*), and a strong correlation between IFN-α, IFN-β, RSAD2, IFI44L, and NMI reemerged after ART initiation.

Our data show that expression of the ISG cluster (RSAD2, ISG15, IFI44L, and IFI27) normalized to near pre-SC levels upon ART initiation.

### Relation Between IFN and ISG Expression and Immune Activation in PWH

**Figure 3.**
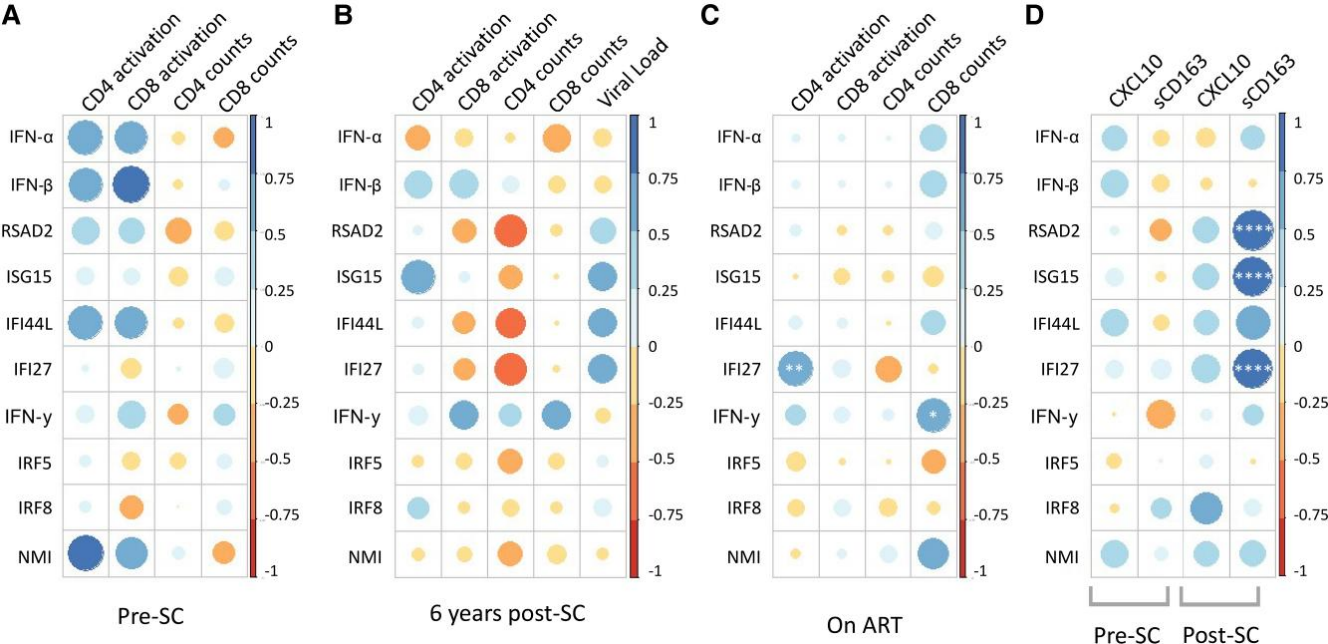
Correlation between interferon-stimulating genes (ISGs) and biomarkers of human immunodeficiency virus disease progression. Depicted are the correlations between ISGs and T-cell activation, T-cell counts, and viral load at pre-seroconversion (SC; *A*), 6 years after SC (*B*), and on antiretroviral therapy (ART; *C*). *D*, Depicted are the correlations between ISGs and serum markers CXCL10 and soluble CD163 at pre- and post-SC. Positive correlations are displayed in blue and negative correlations in red. The color intensity and the size of the circle are proportional to the correlation coefficients. Significant *q* values, as determined by Pearson correlation and adjusted for false discovery rate: *q < 0.05; **q < 0.01; ***q < 0.001; ****q < 0.0001.

Next, we determined whether the IFN and ISG expression levels were associated with biomarkers of disease progression during untreated HIV infection. Positive correlations were shown at pre-SC for both CD4 and CD8 T-cell activation with IFN-α, IFN-β, IFI44L, and NMI (Figure 3*A*). However, this correlation was lost post-SC (Figure 3*B* and Supplementary Figure 4). At 6 years post-SC, there was a positive correlation between CD4 T-cell activation and ISG15 expression and a positive correlation between CD8 T-cell activation and IFN-γ (Figure 3*B*).

Absolute CD4 T-cell counts were negatively correlated with RSAD2, IFI44L, and IFI27 expression levels at 6 years post-SC (Figure 3*B*). Absolute CD8 T-cell counts were positively correlated with RSAD2 at 5–9 months post-SC (Supplementary Figure 4*A*) and IFN-γ levels at 6 years post-SC (Figure 3*B*), whereas a negative correlation with IRF8 levels was observed at 2 years post-SC (Supplementary Figure 4*B*).

The plasma viral load was associated with increased levels of a cluster of ISGs (RSAD2, ISG15, IFI44L, and IFI27), which was statistically significant for IFI27 5–9 months post-SC (Supplementary Figure 4*A*) and IFI44L and IFI27 2 years post-SC (Supplementary Figure 4*B*). Moreover, plasma viral load was positively correlated with IRF5 expression at 2 years post-SC (Supplementary Figure 4*B*).

Correlations between ISG expression levels and biomarkers of HIV disease progression at the pre-ART time point (Supplementary Figure 4*C*) were similar to the 6 years post-SC time point (Figure 3*B*). These correlations were lost after ART initiation (Figure 3*C*). However, a positive correlation between CD4 T-cell activation and IFI27, as well as CD8 T-cell counts and IFN-γ and NMI, was observed on ART (Figure 3*C*).

Soluble biomarkers of inflammation, CXCL10 and sCD163, were measured in serum pre- and post-SC. A strong positive correlation between sCD163 levels and RSAD2, ISG15, IFI44L, and IFI27 was observed at 5–9 months post-SC (Figure 3*D*). Similar correlations between these ISGs and CXCL10 levels were observed, although this did not reach statistical significance. Additionally, IRF8 showed a positive correlation with CXCL10 serum levels post-SC.

Here we show that expression of the ISG cluster (RSAD2, ISG15, IFI44L, and IFI27) was mainly associated with sCD163, and to a lesser extent CXCL10, in serum shortly after seroconversion. Moreover, a correlation with lower CD4 T-cell counts during the progressive course of untreated HIV infection was observed.

### Predictive Value of HIV-Induced IFN and ISG Expression for Disease Progression

**Figure 4.**
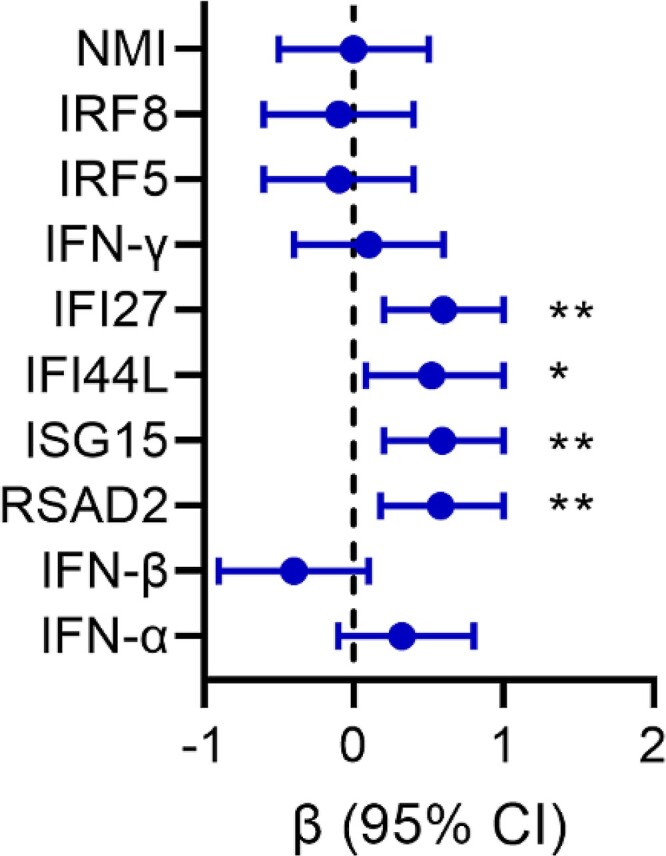
Predictive value of interferons (IFNs) and interferon-stimulating gene (ISG) expression for pre–antiretroviral therapy (ART) viral load. Depicted are the β-coefficient and 95% confidence interval (CI) for IFN and ISG expression levels in people with human immunodeficiency virus at 5–9 months post-seroconversion for viral load before the start of ART (pre-ART). All analyses were corrected for age at seroconversion. Significant *P* values, as determined by linear regression analysis: *P < .05; **P < .01.

Linear regression analysis revealed that expression levels of RSAD2, ISG15, IFI44L, and IFI27 at 5–9 months post-SC were predictive of viral load at pre-ART (Figure 4; Supplementary Table 1), but no significant associations were found between ISGs and pre-ART CD4 T-cell counts (Supplementary Table 2).

Post-SC expression levels of RSAD2, ISG15, and IFI44L were also predictive of CD4 T-cell activation levels at pre-ART (*P* = .015, *P* = .023, and *P* = .014, respectively; Supplementary Table 3), whereas no significant associations were found between ISGs and pre-ART CD8 T-cell activation (Supplementary Table 4).

Our data suggest that expression levels of ISG cluster (RSAD2, ISG15, IFI44L, and IFI27) are predictive for disease outcome.

## DISCUSSION

Here we analyzed type I IFN and ISG expression profile in PBMCs from untreated PWH over the course of infection and compared it to pre-SC levels of the same individuals. After HIV-1 infection, upregulation of a cluster of ISGs (RSAD2, ISG15, IFI44L, and IFI27), as compared to pre-SC, emerged and the expression of these ISGs remained high during the untreated course of infection. This is in accordance with a previous study that showed that these ISGs were persistently upregulated in PWH over 2 years of follow-up [38] and in SIV-infected macaques during the chronic phase of infection [22, 24]. The persistent upregulation of ISGs has been associated with the pathogenic outcome of SIV infection [22, 37, 39]. Here we observed that the ISG cluster was indeed correlated with lower CD4 T-cell counts 6 years after seroconversion, and trends toward higher HIV-1 viral load and CXCL10 levels were observed, confirming previous observations by Mackelprang and colleagues [38]. Expression levels of RSAD2, ISG15, IFI44L, and IFI27 were strongly correlated to serum sCD163 levels early after infection, but not to T-cell activation, suggesting that ISG expression may be linked to monocyte activation. The expression of these ISGs normalized to near pre-SC levels upon ART initiation, suggesting that viral replication may be an important driver of the observed ISG expression.

We observed that type I IFN (IFN-α and IFN-β) expression levels were decreased in the first 2 years after seroconversion as compared to pre-SC levels. Previously, it has been reported that a type I IFN response is induced in the initial phase after infection [27, 37]. In SIV-infected African green monkeys, a strong temporary type I IFN response as determined by plasma levels of IFN-α and IFN-β within weeks after infection is seen [37]. Transcriptome profiling suggested that the type I IFNs were mainly produced in peripheral lymph nodes [37]. The decreased IFN-α and IFN-β expression observed in our study can be explained by the relatively late sampling, 5–9 months after seroconversion, and the use of PBMCs instead of lymph node tissue. Moreover, IFN-α and IFN-β levels normalized to pre-SC levels over the course of infection in at least part of the PWH. Plasma IFN-α levels have previously been positively correlated with plasma viral load and markers for immune activation and inversely correlated with CD4 T-cell counts [40]. Here we observed that IFN-α and IFN-β expression levels were not correlated with biomarkers of disease progression like CD4 T-cell count, viral load or peripheral T-cell activation during the course of infection. This suggests that type I IFNs in plasma are likely produced in peripheral lymph nodes or at other tissue sites by cell such as pDCs, which are major producers of IFN [30, 41, 42]. Moreover, low IFN-α and IFN-β levels in PBMCs could also be explained by the ability of HIV to effectively block IFN production, while it directly induces ISGs [18, 43].

Of the ISGs IFN-γ, IRF5, NMI, and IRF8, only the expression of IFN-γ changed during the course of infection. IFN-γ expression was increased at 6 years post-SC, and levels significantly correlated with CD8 T-cell activation as well as absolute CD8 T-cell counts. This suggests that, consistent with literature, CD8 T cells persistently express IFN-γ during chronic infection and contribute to a proinflammatory environment [37, 44]. The correlation between the IFN-γ expression levels and CD8 T-cell counts remained after ART initiation and may be a reflection of the higher CD8 T-cell counts.

In PWH, we observed that the expression levels of type I IFN and ISG normalized to near pre-SC levels after ART initiation while T-cell activation levels remained significantly elevated. This suggests that ISG induction and T-cell activation are at least in part driven by different mechanisms. Indeed, residual T-cell activation levels after ART initiation have been associated to immune recovery through thymic output and homeostatic proliferation [45, 46]. However, it should be noted that the expression levels of the ISG cluster RSAD2, ISG15, IFI44L, and IFI27 was highly variable and increased expression was observed in part of PWH on ART. The strong correlation between the expression of this ISG cluster and serum sCD163 indicated that the ISG expression may be related to monocyte activation. Monocyte activation has been associated with an increased risk for non-AIDS comorbidities in PWH [29, 47–50]. Activated monocytes show an increased migratory ability [51–53], and augmented migration over of the blood-brain barrier or into the arterial wall has been linked to neuroinflammation and atherosclerosis in PWH [54–60]. Expression levels of these ISGs in PWH on ART could possibly be indicative for an increased risk for non-AIDS comorbidities and identify those who would benefit from intervention therapies to reduce monocyte activation and ISG levels. Indeed, Zhen et al showed in a humanized mouse model that ISG levels reduced after IFN receptor blockage reduced ISG expression even during ART [36].

Most studies analyzing the effect of HIV-1 on IFN and ISG induction use uninfected controls for comparison, or animal models like nonhuman primates infected with SIV or humanized mouse infected with HIV-1. We used pre-SC samples to analyze the effect of HIV-1 infection on IFN and ISG expression, and obtained longitudinal samples from the same individuals during the course of infection and after ART initiation. This minimizes the chance that the observed differences in the expression levels are caused by confounding factors that are different between the study populations like coinfections, age, and lifestyle, which have previously been observed to affect the immune system [8, 54, 61].

Our study has several limitations. The IFN and ISG expression levels were only measured in PBMCs and not in lymphoid tissues. Differences in IFN and ISG expression between tissues have previously been demonstrated [22, 37, 40] and were most prominent in a study by Echebli et al in which IFN-α and IFN-β levels differed between peripheral lymph nodes and PBMCs in African green monkeys infected with SIV [37]. Furthermore, we were unable to distinguish between different cell types and their contribution to ISG production, which could have provided further insights into the mechanisms behind ISG induction. Correlations between ISG cluster (RSAD2, ISG15, IFI44L, and IFI27) and sCD163 levels suggest that monocyte activation may be linked to ISG induction; however, whether peripheral monocytes are the source of the observed ISG induction requires further investigations. The small number of PWH with available pre-SC samples and the limited cell number per sample prevented complete gene expression profiling and we therefore chose to perform a targeted analysis of the IFN and ISG expression profile of genes previously identified in several studies [22, 36–38]. At the time of sampling, a viral load assay with a detection limit of 400 copies/mL has been used and low-level viremia after ART initiation cannot be detected. However, all participants included show a strong decline in viral load on ART and therefore the effect of viral load suppression by ART on ISG levels could be determined. Moreover, only male participants were included in our study, whereas sex differences in ISG expression during HIV-1 infection have previously been reported [62].

Here we performed a longitudinal analysis of IFN and ISG expression in PBMCs before and after seroconversion, during the chronic phase of HIV-1 infection and after ART initiation. We found that HIV-1 infection induced the expression of a set of ISGs (RSAD2, ISG15, IFI44L, and IFI27) that remained upregulated during the chronic phase of HIV-1 infection. The expression of these ISGs strongly associated with sCD163, a biomarker of monocyte activation, and was predictive for disease progression as determined by viral load before ART initiation. The expression levels of these ISGs normalized during ART to pre-seroconversion levels, indicating that the expression of these ISGs is most likely driven by viral replication.

## Supplementary Material

ofae434_Supplementary_Data

## Supplementary Data

Supplementary materials are available at *Open Forum Infectious Diseases* online. Consisting of data provided by the authors to benefit the reader, the posted materials are not copyedited and are the sole responsibility of the authors, so questions or comments should be addressed to the corresponding author.

## Notes

***Author contributions.*** L. v. P. analyzed, interpreted, and visualized data and wrote the manuscript. K. A. v. D., A. F. G., and I. M. performed experiments and analyzed data. A. M. H. provided samples and data. N. A. K. designed research study and experiments, interpreted data, wrote the manuscript, supervised, and acquired funding.

***Acknowledgments.*** We thank all participants in the Amsterdam Cohort Studies (ACS) for their contribution. The ACS on HIV infection and AIDS, a collaboration between the Public Health Service Amsterdam, the Amsterdam University Medical Center of the University of Amsterdam, Medical Center Jan van Goyen, and the HIV Focus Center of the DC-Clinics, is part of the Netherlands HIV Monitoring Foundation and financially supported by the Center for Infectious Disease Control of the Netherlands, National Institute for Public Health and the Environment.

***Financial support.*** This work was supported by AIDS Fonds The Netherlands (grant number P-37202).
