# Supplementary material for: Human Immunodeficiency Virus–Induced Interferon-Stimulated Gene Expression Is Associated With Monocyte Activation and Predicts Viral Load
Source: Open Forum Infect Dis. 2024 Aug 5;11(8):ofae434. doi: 10.1093/ofid/ofae434 (PMC11298257; doi:10.1093/ofid/ofae434)
Supplement: ofae434_Supplementary_Data [file ofae434_supplementary_data.pdf]

Supplementary data

To manuscript: HIV induced Interferon Stimulated Gene expression associates with monocyte activation and predicts viral load

Lisa van Pul[1,2], Karel A. van Dort[1,2], Arginell F. Girigorie[1,2], Irma Maurer[1,2] Agnes M. Harskamp[1,2] & Neeltje A. Kootstra[1,2]
[1]Amsterdam Institute for Infection and Immunity, Amsterdam, The Netherlands
[2]Amsterdam UMC, University of Amsterdam, Experimental Immunology, Meibergdreef 9, Amsterdam, The Netherlands

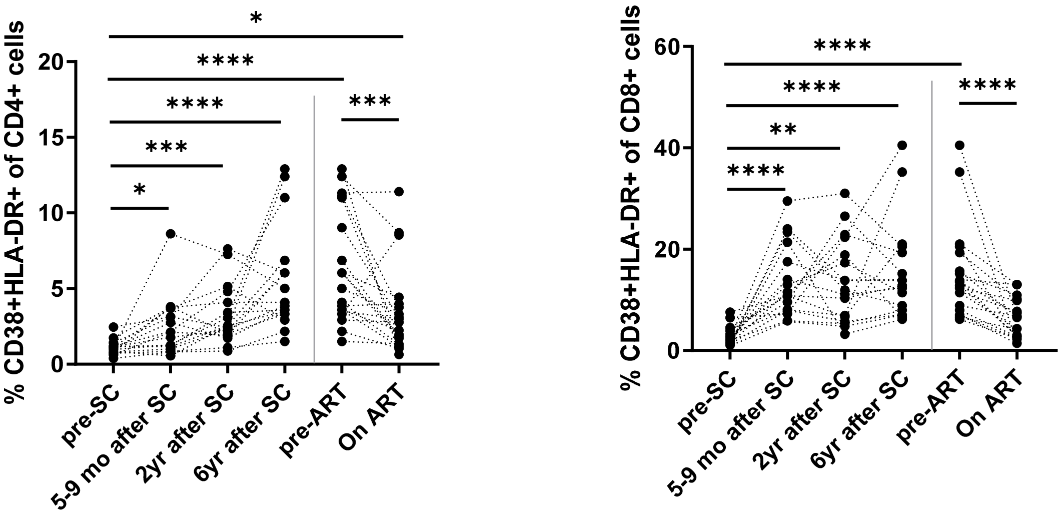

Supplementary figure 1: Longitudinal T cell activation in people with HIV (PWH). CD4 activation (A) and CD8 activation (B), as determined by co-expression of CD38 and HLA-DR, were determined in PWH at pre-seroconversion (SC), at approximately 5-9 months post-SC, 2 and 6 years post-SC. Significant P-values, as determined by Friedman ANOVA test for repeated measurements within an individual followed by Dunn's post hoc tests, are indicated by asterisk: *:P<0.05; **:P<0.01; ***: P<0.001; ****:P<0.0001

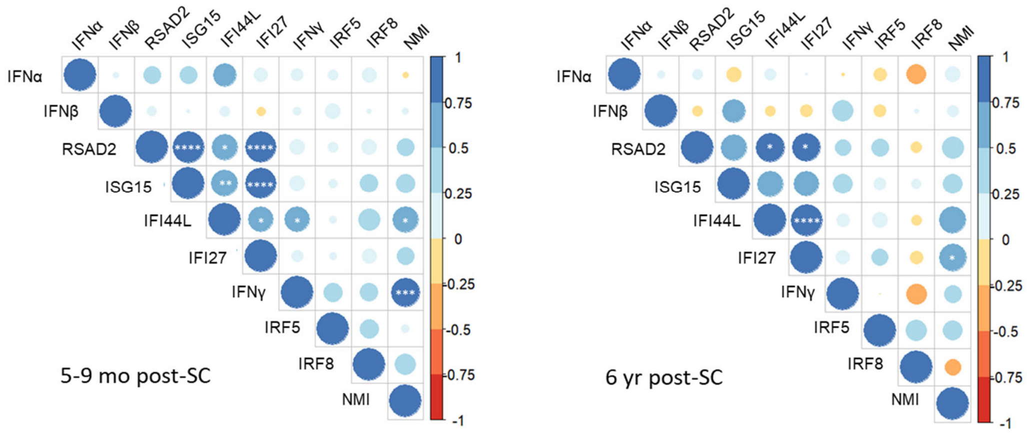

Supplementary figure 2: Correlograms of IFN and ISG expression levels before and during HIV infection. Depicted are the correlations between IFN and ISG expression levels in people with HIV at 5-9 months post-SC (left panel) and 6 years post-SC (right panel). Positive correlations are displayed in blue and negative correlations in red. The color intensity and the size of the circle are proportional to the correlation coefficients. Significant q-values, as determined by Pearson correlation and adjusted for false discovery rate, are indicated by asterisk: *:q<0.05; **:q<0.01; ***:q<0.001; ****:q<0.0001

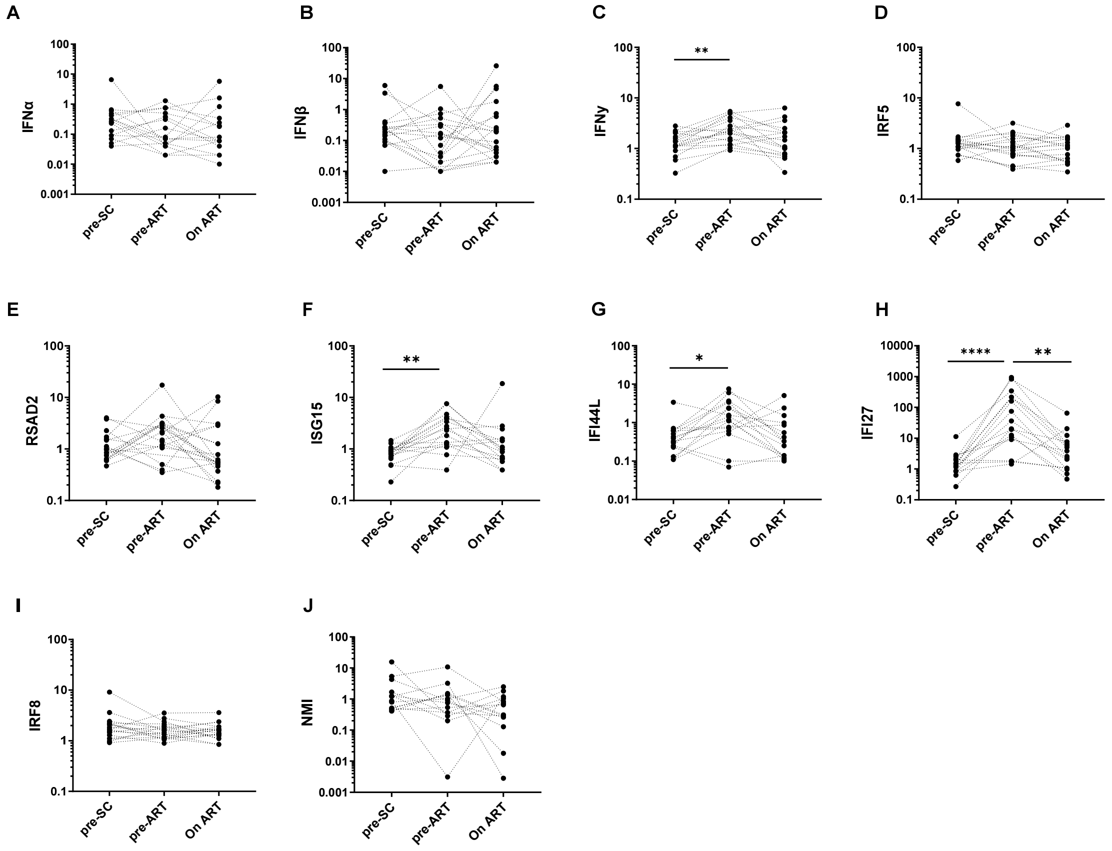

Supplementary figure 3: IFN and ISG expression levels in PWH on ART. Depicted are the IFN and ISG expression levels before HIV infection (pre-SC), and before and after ART initiation. Type I IFNs: IFNα (A) and IFNβ (B); Type I induced ISGs IFNγ (also a type II IFN; C) and IRF5 (D); Type I and type II induced ISGs RASD2 (E), ISG15 (F), IFI44L (G) and IFI27 (H); Type II induced ISGs IRF8 (I) and NMI (J). Expression levels of ISGs were calculated using the $2^{-\Delta\Delta Ct}$ method relative to controls without HIV. Significant P-values, as determined by Friedman ANOVA test for repeated measurements within an individual followed by Dunn's post hoc tests, are indicated by asterisk: *:P<0.05; **:P<0.01; ***:P<0.001; ****:P<0.0001

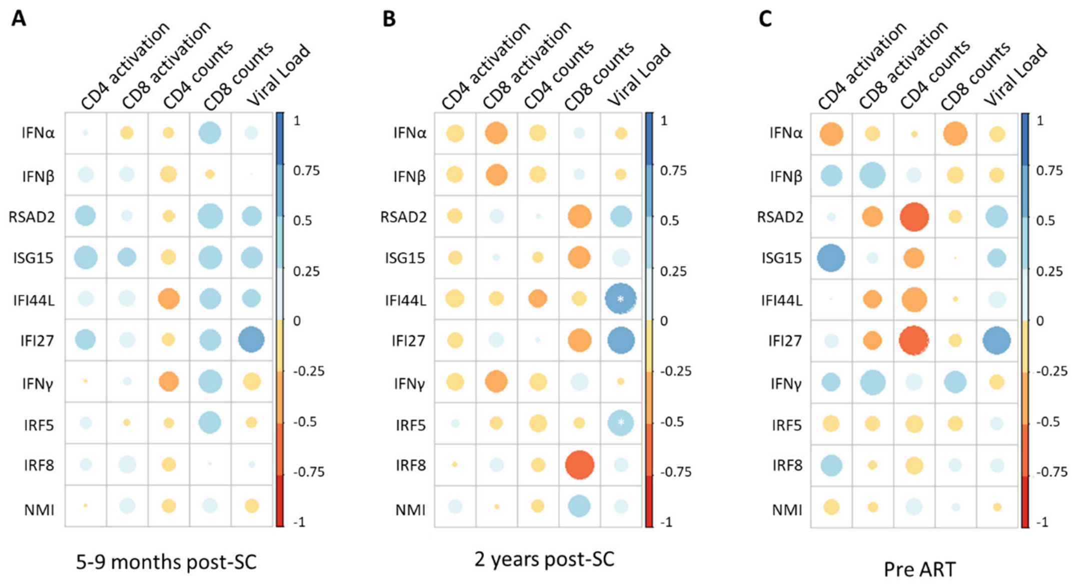

Supplementary figure 4: Correlation between ISGs and biomarkers of HIV disease progression. Depicted are the correlations between ISGs and T cell activation, T cell counts and viral load at 5-9 months (**A**) and 2 years after SC (**B**) and pre-ART (**C**). Positive correlations are displayed in blue and negative correlations in red. The color intensity and the size of the circle are proportional to the correlation coefficients. Significant q-values, as determined by Pearson correlation and adjusted for false discovery rate, are indicated by asterisk: *:q<0.05.

Supplementary table 1: Predictive value of post seroconversion IFNs and ISGs expression for pre-ART viral load

		Univariable analysis	
		**β (95% CI)**	***P***
**IFNα**			
	5-9 months post-SC	0.32 (-0.13 to 0.78)	0.150
**IFNβ**			
	5-9 months post-SC	-0.36 (-0.85 to 0.13)	0.135
**RSAD2**			
	5-9 months post-SC	0.58 (0.18 to 0.97)	**0.007**
**ISG15**			
	5-9 months post-SC	0.59 (0.20 to 0.97)	**0.005**
**IFI44L**			
	5-9 months post-SC	0.52 (0.08 to 0.96)	**0.023**
**IFI27**			
	5-9 months post-SC	0.60 (0.20 to 1.00)	**0.006**
**IFNγ**			
	5-9 months post-SC	0.10 (-0.39 to 0.56)	0.662
**IRF5**			
	5-9 months post-SC	-0.09 (-0.56 to 0.37)	0.674
**IRF8**			
	5-9 months post-SC	-0.11 (-0.58 to 0.35)	0.613
**NMI**			
	5-9 months post-SC	-0.03 (-0.52 to 0.45)	0.889

CI: Confidence Interval.

Supplementary table 2: Predictive value of post seroconversion IFNs and ISGs expression for pre-ART CD4 T cell count

		Univariable analysis	
		**β (95% CI)**	***P***
**IFNα**			
	5-9 months post-SC	0.183 (-0.26 to 0.63)	0.395
**IFNβ**			
	5-9 months post-SC	0.05 (-0.44 to 0.54)	0.830
**RSAD2**			
	5-9 months post-SC	-0.18 (-0.65 to 0.28)	0.413
**ISG15**			
	5-9 months post-SC	-0.14 (-0.61 to 0.33)	0.531
**IFI44L**			
	5-9 months post-SC	-0.03 (-0.52 to 0.46)	0.897
**IFI27**			
	5-9 months post-SC	-0.23 (-0.70 to 0.24)	0.310
**IFNγ**			
	5-9 months post-SC	0.01 (-0.44 to 0.45)	0.984
**IRF5**			
	5-9 months post-SC	0.17 (-0.26 to 0.60)	0.424
**IRF8**			
	5-9 months post-SC	0.22 (-0.21 to 0.64)	0.301
**NMI**			
	5-9 months post-SC	0.001 (-0.45 to 0.46)	0.995

CI: Confidence Interval.

Supplementary table 3: Predictive value of post seroconversion IFNs and ISGs expression for pre-ART CD4 T cell activation[A]

	Univariable analysis	
	**β (95% CI)**	***P***
**IFNα**		
5-9 months post-SC	0.31 (-0.20 to 0.81)	0.218
**IFNβ**		
5-9 months post-SC	0.13 (-0.44 to 0.70)	0.634
**RSAD2**		
5-9 months post-SC	0.50 (0.01 to 0.98)	**0.045**
**ISG15**		
5-9 months post-SC	0.50 (0.02 to 0.98)	**0.041**
**IFI44L**		
5-9 months post-SC	0.57 (0.08 to 1.06)	**0.024**
**IFI27**		
5-9 months post-SC	0.37 (-0.15 to 0.90)	0.153
**IFNγ**		
5-9 months post-SC	0.21 (-0.29 to 0.72)	0.380
**IRF5**		
5-9 months post-SC	-0.01 (-0.52 to 0.51)	0.979
**IRF8**		
5-9 months post-SC	-0.13 (-0.64 to 0.37)	0.583
**NMI**		
5-9 months post-SC	0.13 (-0.39 to 0.66)	0.596

[A] % activated CD4 T cells was log transformed to obtain normal distribution; CI: Confidence Interval.

Supplementary table 4: Predictive value of post seroconversion IFNs and ISGs expression for pre-ART CD8 T cell activation[A]

	Univariable analysis	
	**β (95% CI)**	***P***
**IFNα**		
5-9 months post-SC	0.14 (-0.35 to 0.62)	0.556
**IFNβ**		
5-9 months post-SC	0.33 (-0.18 to 0.83)	0.187
**RSAD2**		
5-9 months post-SC	0.14 (-0.36 to 0.65)	0.559
**ISG15**		
5-9 months post-SC	0.12 (-0.38 to 0.63)	0.611
**IFI44L**		
5-9 months post-SC	0.02 (-0.51 to 0.55)	0.940
**IFI27**		
5-9 months post-SC	0.07 (-0.45 to 0.58)	0.792
**IFNγ**		
5-9 months post-SC	-0.14 (-0.61 to 0.33)	0.532
**IRF5**		
5-9 months post-SC	0.25 (-0.20 to 0.71)	0.258
**IRF8**		
5-9 months post-SC	-0.11 (-0.58 to 0.36)	0.619
**NMI**		
5-9 months post-SC	-0.07 (-0.56 to 0.42)	0.761

[A] % activated CD8 T cells was log transformed to obtain normal distribution; CI: Confidence Interval.

Supplementary table 5: Primer sequences

Target	Accession nr	Forward 5’-3’	Reverse 5’-3’
IFNα	NM_024013.3	GCTTTACTGATGGTCCTGGTGGTG	GAGATTCTGCTCATTTGTGCCAG
IFNβ	NM_002176.4	GAATGGGAGGCTTGAATACTGCCT	TAGCAAAGATGTTCTGGAGCATCTC
ISG15	NM_005101.4	ACACCTGGAATTCGTTGCC	CATCTTTGCCAGTACAGGAGCT
RSAD2	NM_080657.5	TTCCGCTCTACCAATCCAGCT	AAGTCCATCCTGCATGTTGGTG
IFI27	NM_001130080.3	AGAGTCCAGTTGCTCCCAGTGA	TAGCAGCCAAGATGATGTCCG
IFI44L	NM_006820.4	TGCCTACACTGCACTTCCTGTC	ATGTTCAGCTGTACCCTCCCAC
IFNγ	NM_000619.3	AATGTCCAACGCAAAGCAGT	ATTGGGATGCTCTTCGACCT
IRF5	NM_032643.5	ATGCTGCCTCTGACCGA	GCCGAAGAGTTCCACCTG
IRF8	NM_001363907.1	AGCCTTCTGTGGACGATTAC	CTGGGAGAATGCTGAATGGT
NMI	NM_004688.3	CTCGGATCTAGAACCATGGAAGCT	CTGGATCCCTATTCTTCAAAGTATGCTA
GAPDH	NM_002046.7	GGCATGGACTGTGGTCATGA	TGCACCACCAACTGCTTAGC
β-ACTIN	NM_001101.5	GGTCTCAAACATGATCTGGG	GGGTCAGAAGGATTCCTATG
